# An aggregation of human embryonic and trophoblast stem cells reveals the role of trophectoderm on epiblast differentiation

**DOI:** 10.1111/cpr.13492

**Published:** 2023-05-17

**Authors:** Xulun Wu, Wentao Zhao, Hao Wu, Qiancheng Zhang, Yiming Wang, Kunyuan Yu, Jinglei Zhai, Fan Mo, Meijiao Wang, Shiwen Li, Xili Zhu, Xiaoyan Liang, Baoyang Hu, Guang‐Hui Liu, Jun Wu, Hongmei Wang, Fan Guo, Leqian Yu

**Affiliations:** ^1^ State Key Laboratory of Stem Cell and Reproductive Biology, Institute of Zoology Chinese Academy of Sciences Beijing China; ^2^ Institute for Stem Cell and Regeneration Chinese Academy of Sciences Beijing China; ^3^ Beijing Institute for Stem Cell and Regenerative Medicine Beijing China; ^4^ University of Chinese Academy of Sciences Beijing China; ^5^ Reproductive Medicine Research Center The Sixth Affiliated Hospital of Sun Yat‐sen University Guangzhou China; ^6^ State Key Laboratory of Membrane Biology Institute of Zoology, Chinese Academy of Sciences Beijing China; ^7^ Department of Molecular Biology The University of Texas Southwestern Medical Center Dallas Texas USA; ^8^ Hamon Center for Regenerative Science and Medicine University of Texas, Southwestern Medical Center Dallas Texas USA; ^9^ Cecil H. and Ida Green Center for Reproductive Biology Sciences The University of Texas Southwestern Medical Center Dallas Texas USA

## Abstract

The interactions between extra‐embryonic tissues and embryonic tissues are crucial to ensure proper early embryo development. However, the understanding of the crosstalk between the embryonic tissues and extra‐embryonic tissues is lacking, mainly due to ethical restrictions, difficulties in obtaining natural human embryos, and lack of appropriate in vitro models. Here by aggregating human embryonic stem cells (hESCs) with human trophoblast stem cells (hTSCs), we revealed the hESCs robustly self‐organized into a unique asymmetric structure which the primitive streak (PS) like cells exclusively distributed at the distal end to the TS‐compartment, and morphologically flattened cells, presumed to be the extra‐embryonic mesoderm cells (EXMC) like cells, were induced at the proximal end to hTSCs. Our study revealed two potential roles of extra‐embryonic trophectoderm in regulating the proper PS formation during gastrulation and EXMCs induction from the human epiblast.

## Graphical abstract

In this study, an in vitro model (ET‐aggregates, ETAs) was constructed by aggregating human embryonic stem cells (ESCs) and trophoblast stem cells (TSCs) to investigate the crosstalk between human trophectoderm and epiblast. The ES‐compartment of ETAs showed the lumenogenesis and robustly self‐organized into a unique asymmetric structure in which the most of primitive streak‐like cells distributed at the distal end to the TS‐compartment, and morphologically flattened cells, presumed to be the extra‐embryonic mesoderm cells (EXMCs), were induced at the proximal end to hTSCs.

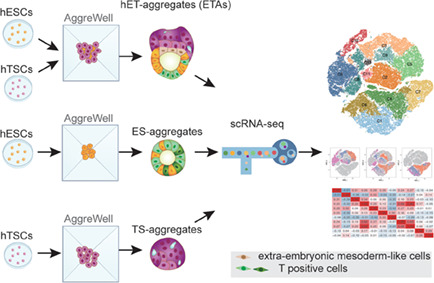

## 1 INTRODUCTION

In early mammalian embryo development, three lineages contribute to the formation of the embryo, including the embryonic epiblast (EPI) which will generate the future organism, and two extra‐embryonic tissues, primitive endoderm (PE, or hypoblast) and trophectoderm (TE), which will form the yolk sac and placenta. These extraembryonic tissues not only are necessary for nutrition but also play crucial roles in regulating embryo development before and during gastrulation.^1^

The previous studies on embryonic and extra‐embryonic tissue crosstalk were almost exclusively conducted in mice, in terms of interactions between the TE and EPI, and it has been reported that the mice polar TE, parts of TE that are located nearest to the EPI, affect the EPI differentiation and proliferation by regulating the NODAL signal. The mouse polar TE expresses and secretes the convertases to cleave the precursor protein of NODAL expressed by EPI, thus forming the full functional NODAL, and causing increased proliferation of EPI. The activated NODAL also diffuses into the adjacent polar TE cells to maintain convertase expression and the Bone Morphogenetic Protein 4 (BMP) transcription. BMP4 from the polar TE then diffuses into the epiblast to induce transcription of the cofactors of NODAL and WNT,^2^, ^3^ and further activate the gastrulation genes such as Brachyury (or TBXT, or T).^4^ However, due to ethical restrictions and lacking research samples, the interactions between embryonic tissues and extra‐embryonic tissues in human embryos are still largely unknown.^5^

Recently, the in vitro embryo‐like structures constructed using the mouse or human stem cells offer us an unique opportunity to study the mammalian embryo development in vitro.^6^ In mice, researchers have successfully created embryo‐like models for the peri‐implantation and post‐implantation stages by combining mouse ESCs with extra‐embryonic stem cells such as TSCs and extra‐embryonic endoderm cells (XENs).^7^, ^8^ These models, known as ETX‐ or ETS‐embryos, can replicate several key spatial and temporal events that occur during embryo development. By incorporating extra‐embryonic cells, these in vitro models offer several advantages over ESCs alone, especially in the context of modelling embryogenesis during gastrulation. These models can capture important developmental events such as primordial germ cell (PGC) specification, PS formation, symmetry breaking, axis formation, and more.^7^, ^8^, ^9^, ^10^ Those findings confirmed the significant role of extra‐embryonic tissues during early development.

Here, we generate a 3D model by aggregating hESCs with hTSCs (ET‐aggregate) to investigate the crosstalk between human trophectoderm and EPI. We found the lumenogenesis, symmetry breaking, asymmetric differentiation of PS‐like cells, and EXMC‐like cell induction in the ES‐compartments under the interaction with hTSCs.^11^, ^12^, ^13^

## 2 MATERIALS AND METHODS

### 2.1 Ethical considerations

The study was approved by the Research Ethics Committee (Research licence 2019SZZX‐008) of Sixth Affiliated Hospital of Sun Yat‐Sen University.

### 2.2 Cell lines and cell culture

The H9 hESC small colonies were plated into 6‐well plates pre‐coated with 1% Matrigel (Corning, 354277) in Essential 8 Medium (E8, Gibco, A1516901) supplemented with 3 μM Y‐27632 (ROCK inhibitor) at 37°C (5% CO_2_). From the second day, hESCs were replaced with fresh E8 every day. hESC colonies were passaged every 5–7 days and dissociated into small colonies by incubation with 0.5 mM EDTA at 37°C for 3 min.^14^, ^15^, ^16^

The hTSCs were derived from our group previously,^17^ and derivation and culture of hTSCs from human embryos were performed as previously described.^18^ hTSCs were maintained in TS complete medium which consists of TS basal medium (DMEM/F12 supplemented with 0.1 mM 2‐mercaptoethanol, 0.2% foetal bovine serum [FBS, Gibco], 0.5% Penicillin–Streptomycin, 0.3% bovine serum albumin [BSA], 1% ITS‐X supplement, 1.5 μg/mL L‐ascorbic acid, 50 ng/mL EGF) and 2 μM CHIR99021, 0.5 μM A83‐01, 1 μM SB431542, 0.8 mM VPA and 5 μM Y27632. After being cultured for 3–4 days, hTSCs around 70%–80% confluency were dissociated with TrypLE Express (Gibco, 12605010) for 8 min at 37°C. The single cells were passaged at a ratio of 1:5 into the 6‐well plates, pre‐coated with 5 μg/mL Collagen I at 37°C for at least 1 h, with TS complete medium.

### 2.3 Generation of ETAs

AggreWell 400 plate (STEMCELL Technologies, 34415) was prepared according to the manufacturer's protocol. Briefly, wells were rinsed with rinsing solution (Stem Cell Technologies, 07010), centrifuged for 5 min at 2000*g* and incubated with rinsing solution at room temperature for 20 min. After incubation, the wells were washed with 2 mL of ×1 Dulbecco's Phosphate Buffered Saline (DPBS), and 500 μL E8 medium with CEPT cocktail (Chroman 1, Emricasan, Polyamine, and trans‐ISRIB)^19^ was added to each well. The plate was spun for 5 min at 2000*g*, then placed at 37°C (5% CO_2_) until use.

The hESC colonies were dissociated to single cells by incubation with Accutase (Invitrogen, A1110501) at 37°C for 8 min, and hESCs were pelleted by centrifugation for 3 min at 1200 rpm. Single cells were resuspended in E8 medium with CEPT cocktail and 14,400 hESCs per well were added in AggreWell plate. There was around 1.5 mL medium per well and 12 cells per microwell. The following day, hTSC colonies were dissociated to single cells by incubation with TrypLE at 37°C for 8 min. hTSCs were pelleted by centrifugation for 4.5 min at 1100 rpm. After removing 500 μL E8 medium each well, hTSC single cells were resuspended in hTSC basal medium with 0.8 mM VPA, and CEPT cocktail and 80,000 hTSC single cells per well were added in AggreWell plate. All hESC and hTSC single cells were counted by a Luna Automated Fluorescence Cell Counter.

The AggreWell plate was then centrifuged for 3 min at 100*g*, and placed at 37°C under 5% CO_2_ condition. The time when hTSCs were added into the plates was designated as 0 h, and aggregates formed after around 12 h of culture. For the first 48 h, hESC and hTSC aggregates were cultured at 37°C (5% CO_2_) in mixed medium. After 48 h, the aggregates were transferred to 40% Matrigel (Corning, 354230) and cultured in medium 1 (APEL2 medium added both 3 μM CHIR99021 and 50 ng/mL EGF). After 12 h, replaced the medium 2 with CHIR99021 removed. The 72 h ETAs were changed to medium 3 (APEL2 added 3 μM CHIR99021, 5 ng/mL FGF2, and 50 ng/mL EGF) until the samples were collected at 96 h.

### 2.4 Generation of ES‐aggregates/TS‐aggregates

Preparation of AggreWell 400 plate was same as above. hESCs or hTSCs will not be added into wells, but the same culture medium will be added. Other steps are the same as generation of ETA.

### 2.5 Immunostaining

ETAs and ES‐aggregates were fixed in 4% PFA for 30 min at room temperature, washed thrice in PBS, and permeabilized for 30 min at room temperature in 0.4% Triton X‐100. After being blocked in blocking buffer (3% bovine serum albumin) for 1 h, the ETA/ES‐aggregates were incubated with primary antibodies diluted in blocking buffer overnight at 4°C. After three washes in PBST (PBS plus 0.2% Tween‐20), ETAs were incubated for 1 h with secondary antibody solution containing DAPI and Phalloidin at room temperature. Finally, ETAs/ES‐aggregates were washed thrice in PBST and then transferred to the maintenance solution (0.05% PBST plus 0.1% BSA) before confocal imaging. Antibodies used for immunofluorescence include:

**Table:** 

Antibodies	Brand	Catalogue	Working dilution
OCT4	Cell Signaling Technology	2750	1:200
OCT‐3/4 (C‐10)	Santa Cruz Biotechnology	sc‐5279	1:200
SOX2 (D6D9)	Cell Signaling Technology	3579	1:200
GATA3 (D13C9)	Cell Signaling Technology	5852	1:200
GATA3	Invitrogen	MA1‐028	1:200
CGB	Abcam	ab53087	1:200
Podocalyxin	R&D Systems	AF‐1658	1:200
Brachyury	Cell Signaling Technology	81694s	1:200
Brachyury	Invitrogen	PA5‐46984	1:200
ISL1	Abcam	ab86472	1:100
AP‐2 alpha Monoclonal	DSHB	3b5	1:10
Phalloidin	YEASEN	40735ES75	1:1000
Donkey Anti‐Mouse IgG (H + L) Alexa Fluor 488	Invitrogen	R37114	1:100
Donkey anti‐Mouse IgG (H + L) Alexa Fluor 568	Invitrogen	A10037	1:100
Donkey anti‐Rabbit IgG (H + L) Alexa Fluor 568	Invitrogen	A10042	1:100
Donkey Anti‐Goat IgG (H + L) Alexa Fluor 488	Invitrogen	A‐11055	1:100
Goat Anti‐Mouse IgG (H + L) Alexa Fluor 647	Invitrogen	A‐21236	1:100

### 2.6 Imaging

All immunofluorescence images were acquired by the ZEISS LSM 880 confocal laser‐scanning microscopes with the ×20 air objectives, or ×40 oil‐immersion objectives. Fluorophores were excited with a 405 nm diode laser (DAPI), a 488 nm argon laser (Alexa Fluor), a 568 nm HeNe laser (Alexa Fluor) and a 647 HeNe laser (Alexa Fluor). Images were acquired with 1–2 μm z separation. For a better presentation, the images of single structure were cropped and angled. All analyses were carried out using open‐source image analysis software including Zeiss LSM Image Browser Software, Imaris Software, and Fiji Image J (NIH).

Three‐dimensional (3D) visualizations of the ETAs/ES‐aggregates were performed using Imaris software. The manual surface rendering module was used for 3D reconstruction of cell nuclei in ETAs and counting cell number of the ES‐compartment. The diameters of the lumen and the ES‐compartment in the ETAs were measured using Imaris point measurement tool. The immunofluorescence intensity of PODXL, SOX2, F‐actin, and T was measured by ZEN software.

### 2.7 Preparation of single‐cell transcriptome library and sequencing

We selected ~500 ETAs, ES‐aggregates, and TS‐aggregates from each of the three‐time points (48, 72, and 96 h) for single‐cell RNA sequencing using the 10× Genomics platform. The morphological selection criteria of 48 h, 72 h, and 96 h ETAs were: (1) hTSCs combined hESCs and wrapped the hESCs under the bright‐field microscope; (2) the diameter of ETAs was 150–230 μm; (3) the presence of a lumen in the embryoids under the bright‐field microscope; (4) Circular, bright and good refraction. All ETAs were manually pooled using a Pasteur pipet, washed three times in ×1 DPBS and dissociated with mixed enzyme 1 (TrypLE: 0.05% Dnase I = 5: 1) in shaking bath at 37°C. PBS (1 mL) containing 5% FBS was then added into the samples and the samples were centrifuged at 1000 rpm for 1 min. Cell pellets were resuspended in PBS containing 0.1% BSA and filtered through a 70‐μm cell strainer. Cell number was determined using a Luna Automated Fluorescence Cell Counter. Single‐cell suspensions (0.5–1 × 10^6^ cells/mL) were loaded into a 10× Genomics Chromium Chip following the manufacturer's instructions (10× Genomics, Chromium Next GEM Single Cell 3′ GEM, Library and Gel Bead Kit v.3.1). As the control, single cells of ES‐aggregates, and TS‐aggregates were collected by a similar strategy.

### 2.8 Quality assessment and preprocessing of single‐cell sequence data

For sequencing data generated by the 10× Genomics platform, raw sequencing reads were aligned to GRCh38, and the count matrix was generated by using Cell Ranger (v6.1.1). The quality control for cells was carried out and cells were included if they met the following criteria: (1) the number of detected genes was between 1500 and 7500; (2) the percentage of mitochondrial gene sequences was <10%.

### 2.9 Downstream analysis of single‐cell RNA sequencing data

The following data analysis of single‐cell RNA sequencing was mainly performed by using Seurat (v4.0.6).^20^ Firstly, raw counts were normalized by using *NormalizeData*, and 2000 high variable genes (HVGs) were identified by using the *FindVariableFeatures* with default parameters. Then, dimensionality reduction was performed by using the combination of *RunPCA* and *RunTSNE* with dims = 1:10, followed the cluster identification by using *FindNeighbors* with dims = 1:10 and *FindClusters* with resolution = 0.5. For integration of the public single‐cell data, *SCTransform* ^21^ was used with the default parameters, and then the correlation among cell types was measured by Pearson correlation based on the average expression levels of genes within the specific cell type, and the integrated expression levels genes were used for correlation calculation.

### 2.10 Identification of differentially expressed genes and GO enrichment analysis

The differentially expressed genes (DEGs) were identified by using the *FindAllMarkers* with the parameters logfc.threshold = 0.25. And GO enrichment analysis were performed by using the R package clusterProfiler (v4.2.1).^22^

### 2.11 Inferring cell–cell communication with single‐cell RNA sequencing data

The normalized expression data from scRNA‐seq were used to infer the cell–cell communication by using CellChat (v1.6.1)^23^ with default parameters and *netAnalysis_signalingRole_heatmap* was used to identify signals contributing most to outgoing or incoming signalling.

### 2.12 Statistical analysis

Values were shown as mean ± SEM. Statistical parameters including statistical analysis and statistical significance reported in the figure legends and supplementary figure legends in Data S1 were obtained using t‐test and ANOVA through GraphPad Prism8. Significance was defined as *P < 0.05; **P < 0.01; ***P < 0.001, ****P < 0.0001.

## 3 RESULTS

### 3.1 Generation of human embryonic stem cells and trophoblast stem cells co‐differentiation aggregates

**FIGURE 1:**
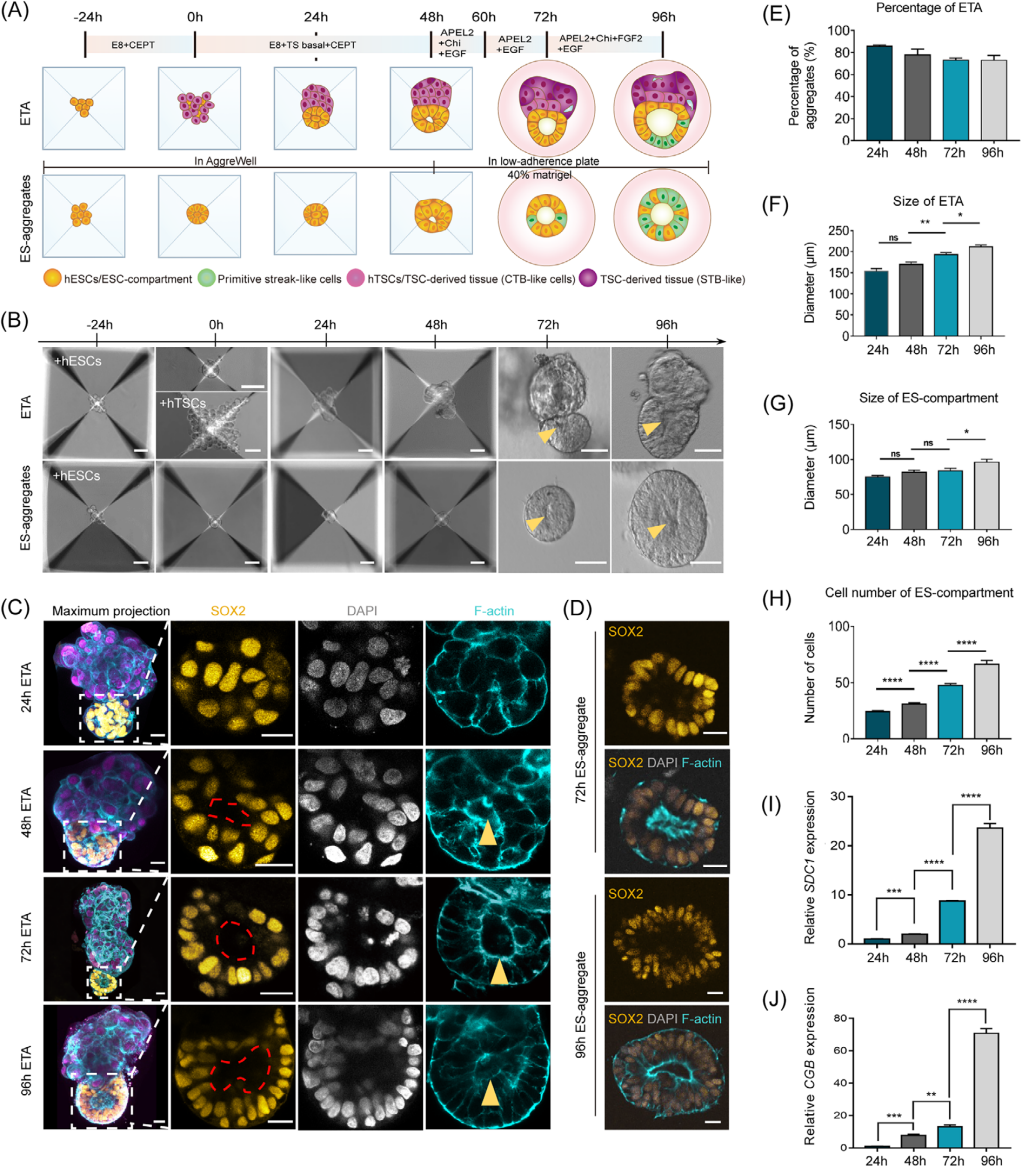
Generation of ETAs using human ESCs and TSCs based on a co‐differentiation system. (A) Scheme of the protocol to generate ETAs, comprising ES‐compartments and TS‐compartments, and ES‐spheroids. hESCs, gold, human embryonic stem cells; hTSCs, violet, human trophoblast stem cells. The dark violet compartment represents multinucleated syncytiotrophoblast (STB). Green cells represent gastrulating cells. E8, Essential 8 medium; CEPT, Chroman 1, Emricasan, Polyamine, and trans‐ISRIB; TS basal, TS basal medium; APEL2, STEMdiff APEL2 Medium; Chi, Chiron (CHIR99021); EGF, epidermal growth factor. (B) Bright‐field images of hESCs and hTSCs plated in the AggreWell plate forming ETAs, and hESCs forming ES‐aggregates. The yellow arrows point to cavities in the ETAs and ES‐aggregates. Scale bars, 50 μm. (C) Representative images of ETAs immunostained for indicated markers. The leftmost panels are maximum projections, and other panels are selected single slice. The red dotted line and yellow arrowhead indicates a cavity in the ES‐compartment of ETAs at 48, 72, and 96 h. SOX2, gold, the marker of hESCs; GATA3, violet, the marker of hTSCs; F‐actin, cyan, the marker of cytoskeletal component; DAPI, grey, DNA, here and after. Scale bars, 20 μm. (D) Representative images of ES‐aggregates immunostained for SOX2 (gold) and F‐actin (cyan). Scale bars, 20 μm. (E) Bar graph showing the proportion of ETAs comprising ES‐ and TS‐compartments at different time points. *n* = 100 ETAs per group, three experiments. (F) Bar graph showing the mean tissue diameter of indicated ETAs. *n* = 10 ETAs per group, three experiments. Data are shown as mean ± SEM. Student's *t* test: ns, not significant; *P < 0.05, **P < 0.01. (G) Bar graph showing the mean tissue diameter of indicated ES‐compartment of ETAs. *n* = 10 ETAs per group, three experiments. Data are shown as mean ± SEM. Student's *t* test: ns, not significant; *P < 0.05. (H) Bar graph showing the mean quantification of ES‐compartment cell numbers in indicated ETAs. *n* = 10 ETAs per group, three experiments. Data are shown as mean ± SEM. Student's *t* test: ****P < 0.0001. (I) qPCR analysis of SDC1 mRNA in 24, 48, 72, and 96 h ETAs, three experiments. Data are shown as mean ± SEM. Student's *t* test: ns, not significant; ***P < 0.001, ****P < 0.0001. (J) qPCR analysis of CGB mRNA in 24, 48, 72, and 96 h ETAs, three experiments. Data are shown as mean ± SEM. Student's *t* test: ns, not significant; **P < 0.01, ***P < 0.001, ****P < 0.0001.

How human embryonic and extra‐embryonic tissues cooperate to ensure normal embryo development is an important question for developmental biology. To investigate the interactions between human epiblast and trophectoderm during early embryo development, we aggregated human embryonic stem cells (hESCs) and human trophoblast stem cells (hTSCs) to generate a 3D co‐differentiation structure, termed ETA (hESCs and hTSCs aggregate, ET‐aggregate). The hESCs were digested into single cells and seeded into microwells of AggreWell 400 in E8 medium supplied with CEPT cocktail to enhance the cell viability,^19^ and the hTSCs were seeded 24 h later. The time of seeding hTSCs was defined as 0 h (Figure 1A,B). After aggregation of hTSCs, the ETA structures were cultured in ET medium (1:1 mixture of E8 and hTSC basal medium supplied with VPA and EGF to boost the growth of both hESCs and hTSCs). At 24 h, over 80% of aggregates self‐organized into a bipolar structure consisting of a hESC polar and a hTSC polar (Figure 1A‐C,E). Then, at 48 h, to mimic a 3D basal membrane that is indispensable for the lumenogenesis and gastrulation of human epiblast, the ETAs were embedded in the Matrigel and cultured in the STEMdiff APEL 2‐based medium (APEL2) for 48 h extended culture. The size of ETAs with the diameter and cell number of ES‐compartment of ETAs was gradually increased during culture periods (Figure 1B,F‐H). Furthermore, we have confirmed that the TS‐compartment of ETAs maintains normal growth and the syncytialization markers *SDC1* and *CGB* were significantly upregulated (Figure 1I,J).

### 3.2 Cavity formation in the ES‐compartments of ETAs

**FIGURE 2:**
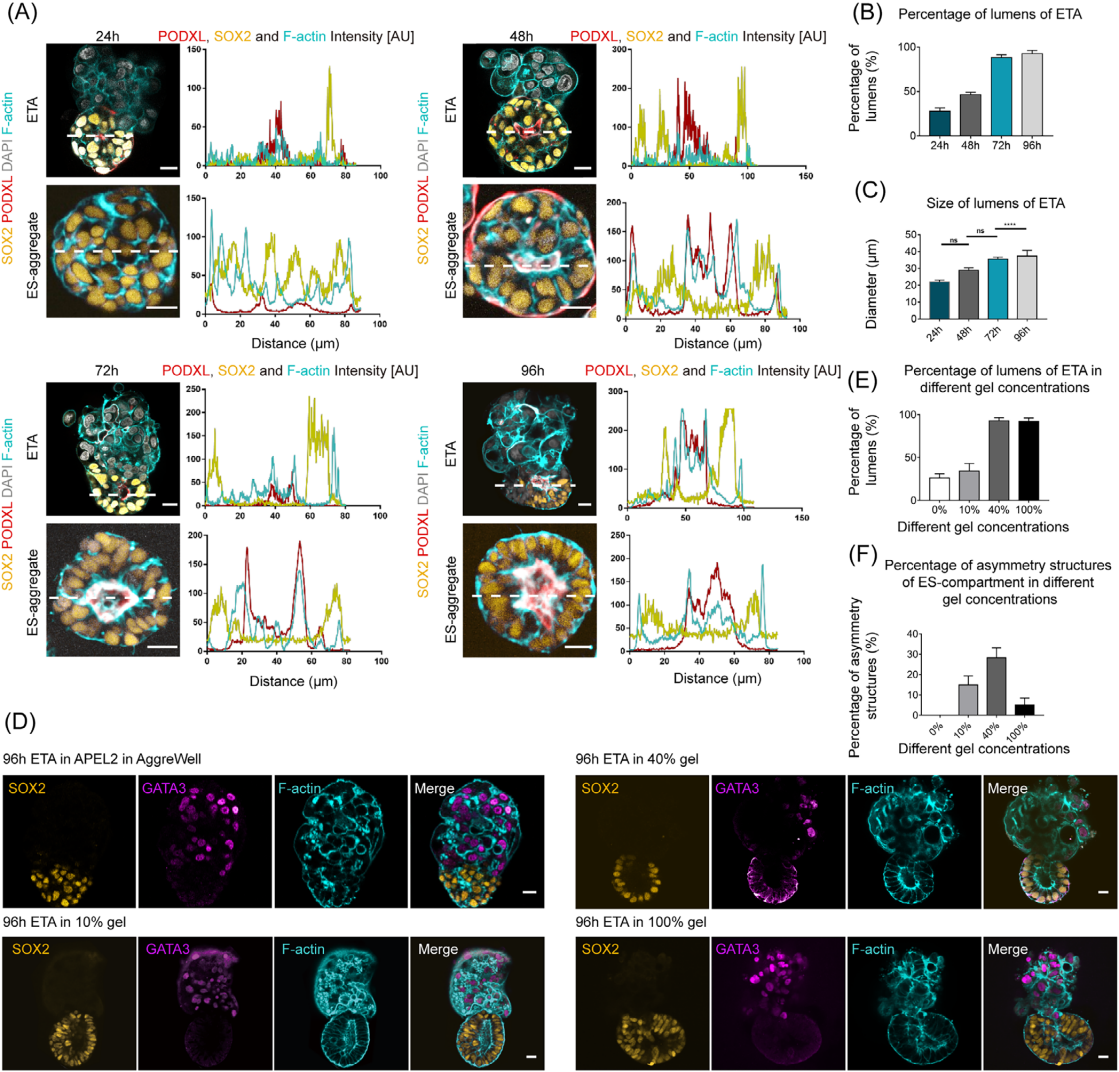
Cavity formation in the ES‐compartments of ETAs. (A) Immunostaining of 24, 48, 72, and 96 h ETAs and ES‐aggregates showing the progression of cavitation using antibodies against SOX2 (gold), F‐actin (cyan), and PODXL (red, the marker of anti‐adhesive sialomucin protein). Intensity scans of PODXL, SOX2, and F‐actin along indicated dashed white lines taken at the middle of ES‐compartments. The accumulation of PODXL on the apical side of SOX2^+^ cells facing a lumen indicates the presence of a cavity, which is indicated by two peaks of PODXL in the intensity profile. DAPI, grey, DNA, here and after. *n* = 10 ETAs per group, three experiments. Scale bars, 20 μm. (B) Bar graph showing the proportion of ES‐compartment of ETAs comprising a lumen. *n* = 100 ETAs per group, three experiments. (C) Bar diagram indicating the mean lumen diameter of the indicated ETAs. *n* = 10 ETAs per group, three experiments. Student's *t* test: ns, not significant; ****P < 0.0001. (D) Representative images of 96 h ETA, which were cultured in AggreWell during the whole culture period (top panels) and transferred into 10% (second panels)/40% (third panels)/100% Matrigel (bottom panels) at 48 h, immunostained for SOX2, GATA3, and F‐actin. (E) Bar graph showing the proportion of ETAs comprising lumens in ES‐compartments at 0%, 10%, 40%, and 100% Matrigel. *n* = 100 ETAs per group, three experiments. (F) Bar graph showing the proportion of ETAs comprising asymmetry structures in ES‐compartments at 0%, 10%, 40%, and 100% Matrigel. *n* = 100 ETAs per group, three experiments.

The apical–basal polarization and lumenogenesis of epiblast were key morphogenetic events in early human embryo development. At 24 h, immunostaining of F‐actin showed that the ES‐compartment of ETAs was robustly self‐organized into a lumen‐like cyst structures with a tiny cavity, which gradually expanded during the following days (Figure 2A‐C). Meanwhile, as a control, we cultured hESCs alone to generate ES‐aggregates under the same condition as ETAs (Figure 1A,B). Similarly, in ES‐aggregates, a central cavity was observed at 48 h and further enlarged with culture (Figure 1B,D).

To evaluate the formation of the central cavity in the ES‐compartments of ETAs, we detected the expression of Podocalyxin (PODXL), a transmembrane protein, and the accumulation of PODXL along the apical side was observed at 24 h, which significantly delineated a lumen at 48 h (Figure 2A). Correspondingly, with the increase of culture time, formation efficiency (Figure 2B) and average diameter (Figure 2C) of the cavity was also increased. At 96 h, the average diameter of the cavity in ES‐compartments was 37.3 μm, and the formation efficiency was about 92.7% (Figure 2B,C).

Besides, the basement membrane is important for lumenogenesis in ETAs. To determine the most appropriate Matrigel concentration for lumenogenesis, we detected the efficiency of lumenogenesis of the ETAs cultured in 10%, 40%, and 100% Matrigel (Figure 2D). We found that the ETAs cultured in 40% Matrigel showed the best cavity formation efficiency of 92.7% (Figure 2E) and the asymmetric structure of ES‐compartment of 28.3% (Figure 2F).

### 3.3 Trophoblast stem cells induced asymmetric differentiation of embryonic stem cells in ETAs

**FIGURE 3:**
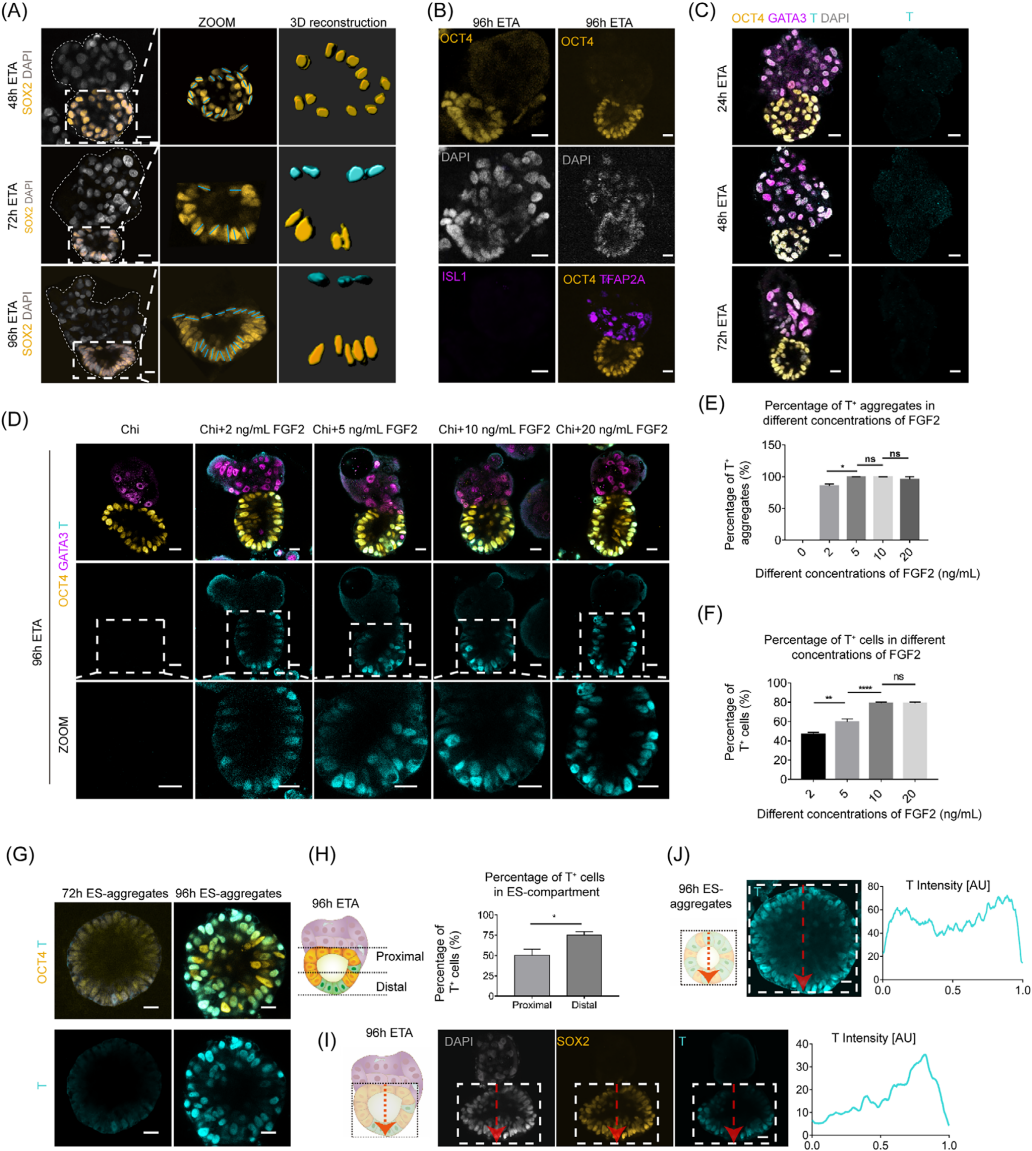
Trophoblast stem cells induced asymmetric differentiation of embryonic stem cells in ETAs. (A) Immunostaining (leftmost panels) and 3D reconstruction images (rightmost panels) of the nuclear shape of ES‐compartment cells showing the columnar (gold) and flattened (cyan) nuclear shapes at 72 and 96 h. Scale bars, 20 μm. (B) Representative images of 96 h ETAs immunostained for OCT4 and amnion cell markers (ISL1 and TFAP2A, purple) showing there was no amnion cells in 96 h ETA. Scale bars, 20 μm. (C) Representative images of ETAs immunostained for indicated markers at 24, 48, and 72 h. OCT4, gold, the marker of hESCs; T, cyan, the marker of primitive streak‐like cells. Scale bars, 20 μm. (D) The representative images of 96 h ETAs which were exposed to 3 μM Chi and 0, 2, 5, 10, and 20 ng/mL FGF2 24 h and stained for OCT4, GATA3, and T. Chi, Chiron (CHIR99021). (E) Bar graph showing the T^+^ aggregates proportion of 96 h ETAs which were exposed to 0, 2, 5, 10, and 20 ng/mL FGF2 24 h. *n* = 10 ETAs per group, three experiments. Student's *t* test: ns, not significant; *P < 0.05. (F) Bar graph showing the proportion of T^+^ cells in the ES‐compartment of 96 h ETAs which were exposed to 0, 2, 5, 10, and 20 ng/mL FGF2 24 h. *n* = 100 ETAs per group, three experiments. Student's *t* test: ns, not significant, **P < 0.01, ****P < 0.0001. (G) The representative images of 72 h ES‐aggregates and 96 h ES‐aggregates which were exposed to 3 μM Chi and 5 ng/mL FGF2 24 h stained for OCT4 and T. (H) The proportion of T^+^ cells in the proximal and distal part in the ES‐compartment of ETA. One half of the ES‐compartment delimits the proximal part (close to TS‐compartment) and distal part (far from TS‐compartment). *n* = 10 ETAs per group, three experiments. Student's *t* test: ns, not significant; *P < 0.05. (I) Immunostaining of 96 h ETAs showing asymmetry distribution of T positive signal (cyan) in the ES‐compartment (SOX2, gold). Distribution of fluorescence signal in the boxed area along the dashed red line is analysed from TS‐compartment to distal regions at the middle of ES‐compartments, showing fluorescence peak appears at the distal part of TS‐compartment. The X‐axis represents the relative distance from proximal to distal part. *n* = 10 ETAs per group, three experiments. (J) Immunostaining of 96 h ES‐aggregates showing symmetry distribution of T positive signal (cyan) in the ES‐compartment (SOX2, gold). Distribution of fluorescence signal in the boxed area along the dashed red line is analysed by random side to side at the middle of ES‐compartments, showing two fluorescence peaks appear at both ends of the relative distance. The X‐axis represents the relative distance from proximal to distal part. *n* = 10 ETAs per group, three experiments.

WNT signalling was known to be important for symmetry breaking of human epiblast and was activated in epiblast during gastrulation.^24^, ^25^, ^26^ To simulate the gastrulation, the ETAs were treated with 3 μM CHIR99021 (a WNT agonist) combined with 5 ng/mL FGF2 at 72 h (Figure 1A), as FGF2 was known to induce the PS formation of the posterior epiblast. After treatment, the ES‐compartment of ETAs obviously differentiated into an asymmetrical structure at 72 h, with a single layer of flattened cells at the pole contact with the TS‐compartment, and a columnar epiblast‐like epithelium at the other pole (Figure 3A). The flattened cells were morphologically similar to the amnion cells, a primate‐specific extra‐embryonic cell type, as previously reported.^27^, ^28^ However, we found these cells were negative for key amnion markers such as *ISL1* ^29^ and *TFAP2A* (transcription factor AP‐2‐alpha),^30^ indicating a non‐amnion identity (Figure 3B). Interestingly, the flattened cells were not seen in the ES‐aggregates cultured in the same condition, and the ES‐aggregates developed into a homogenized epithelium structure (Figures 1B,D and 2A), indicating that the flattened cells were specific to ETAs.

At 96 h, a small population of T‐box transcription factor T (TB , or brachyury, or T) positive cells were exceptionally distributed at the distal part of the epithelium‐compartment of ETAs (Figure 3D,I). Before 96 h, no cells expressed T in ETAs (Figure 3C). In contrast, the T‐positive cells were observed in ES‐aggregates as early as 72 h, and the number of T‐positive cells was significantly increased at 96 h (Figure 3G). However, the T‐positive cells in ES‐aggregates showed random distribution without asymmetry (Figure 3G,J), suggesting that hTSCs may also play roles in regulating the differentiation of epiblast to T‐positive cells.

Next, we evaluated different FGF2 concentrations for the T‐positive induction of ETAs (Figure 3D). Exposed to 0, 2, 5, 10, and 20 ng/mL FGF2, the proportion of ETAs expressing T was 0, 85.4%, 99.3%, 99.4%, and 96.0%, respectively (Figure 3E). The percentage of the T‐positive cells in ETAs exposed to 5 ng/mL FGF2 reached 59.5% (Figure 3F). Thus, 5 ng/mL FGF2 was determined for inducing T‐positive cells in ETAs. The proportion of the ETAs with T‐positive cells at the proximal and distal region of the TS‐compartment was about 50.2% and 75.4%, respectively (Figure 3H). In contrast, the T‐positive cells developed in the ES‐aggregates treated with 5 ng/mL FGF2 showed no asymmetric distribution (Figure 3J). Taken together, hTSCs showed a potential role in the asymmetric differentiation of human epiblast.

### 3.4 Transcriptome characterization of ET‐aggregates at single‐cell resolution

**FIGURE 4:**
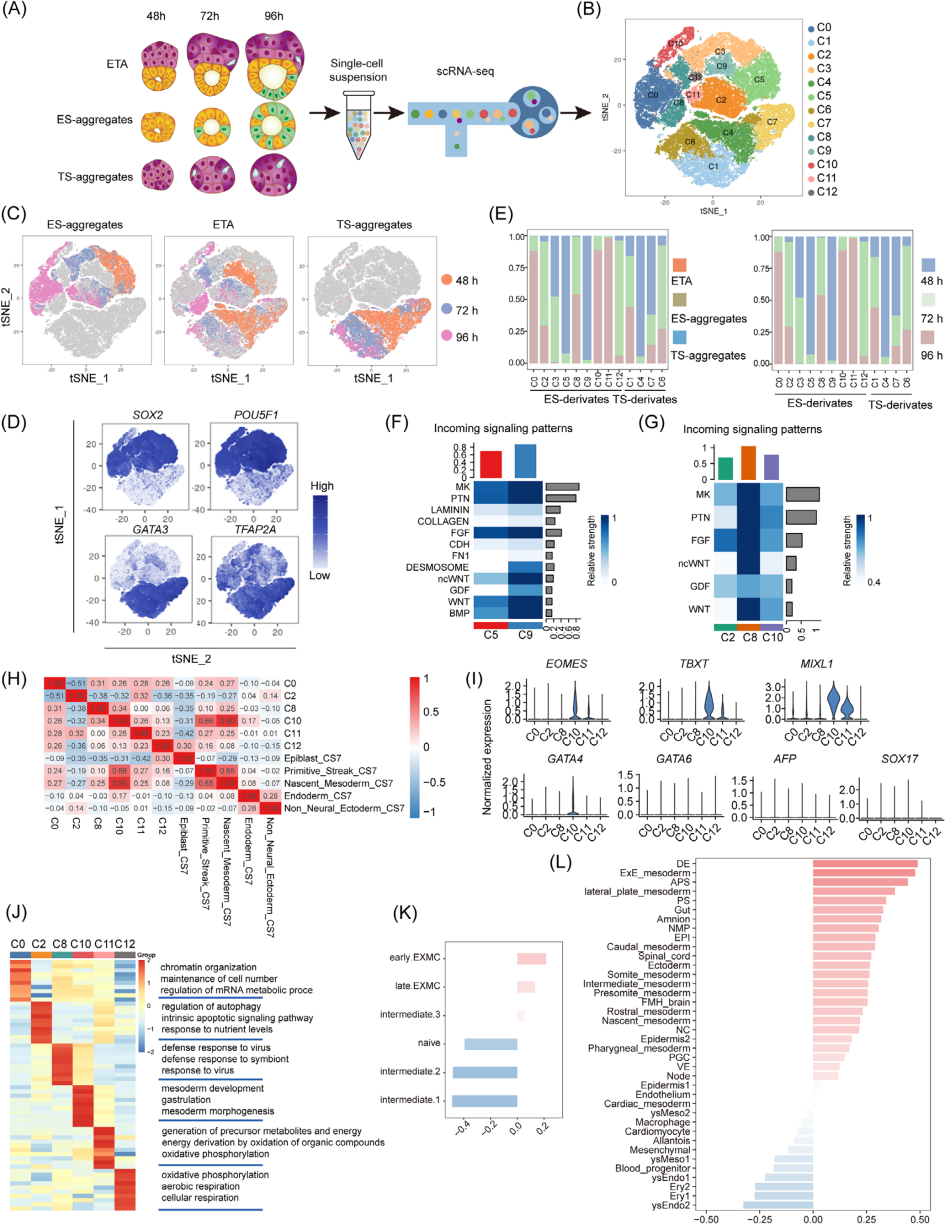
Transcriptome characterization of ETAs, ES‐aggregates, and TS‐aggregates at single‐cell resolution. (A) Scheme of the protocol to generate scRNA‐seq libraries. (B) t‐SNE plot showing 13 cell clusters. (C) t‐SNE plots showing the distribution of timecourse from ES‐aggregates (left), ETAs (middle) and TS‐aggregates (right). (D) t‐SNE plots showing expression levels of ES‐derivates (*SOX2/POU5F1*) and TS‐derivates (*GATA3/TFAP2C*) marker genes. (E) Sample (left) and timepoint (right) distribution of each cell cluster from scRNA‐seq. (F) Heatmap shows the relative strength of cell cluster from 48 h ETAs (C5, C9, and TS‐compartment) based the incoming signalling patterns. (G) Heatmap shows the relative strength of cell clusters 72 h and 96 h ETAs (C2, C8, C10, and TS‐compartment) based the incoming signalling patterns. (H) Correlation analysis of clusters from ES‐compartment and ES‐aggregates with cells from CS7 human embryos. (I) Violinplots showing the expression levels of PS, NM, and endoderm marker genes. (J) Heatmap showing the scaled expression patterns of top 10 genes for clusters from ES‐compartment of ETAs and ES‐aggregates. (K) Correlation analysis of C8 from 72 to 96 h ETAs with EXMCs. (L) Correlation analysis of C8 from 72 h to 96 h ETAs with cells from CS8‐11 monkey embryos.

To further characterize the cell types developed in ETAs, we performed single‐cell RNA sequencing (scRNA‐seq) for the ETAs, ES‐aggregates, and TS‐aggregates at 48, 72, and 96 h using 10× Genomics platform (Figure 4A). By strict filtering, 22,607, 29,998, and 18,996 high‐quality cells were retained from ETAs, ES‐aggregates, and TS‐aggregates, respectively, with more than 6000 genes detected. The uniform manifold approximation and projection (UMAP) analysis identified 13 distinct cell populations (C0–C12) in the three structures at three different time points (Figure 4B). The ES‐derivates and TS‐derivates were mainly separated into two different populations (Figure 4C). The pluripotency markers *SOX2* and *POU5F1* were highly expressed in the ES‐derivates, including clusters 0, 2, 3, 5, 8, 9, 11, and 12. The trophectoderm markers *GATA3* and *TFAP2A* were highly expressed in the TS‐derivates, including clusters 1, 4, 6, and 7 (Figure 4D). Interestingly, compared with the ES‐derivates in ES‐aggregates, the clusters of the ES‐derivates in ETAs were dramatically shifted (Figure 4C), while the clusters of TS‐derivates from both ETAs and TS‐aggregates were generally overlapped (Figure 4C), the dramatic changes in gene expression pattern were also not detected during the differentiation of hTSCs regardless of whether hESCs co‐exist, which suggested an asymmetric interaction between hESCs and hTSCs, and the hTSCs differentiation was not obviously affected by co‐culture with hESCs. Therefore, we will mainly focus on the ES‐derivates herein.

Firstly, we identified two unique clusters C8 and C9 were specific to the ES‐compartment of 72–96 h and 48 h ETAs, respectively (Figure 4E). We next used the CellChat^23^ to investigate the cell–cell communication between clusters. As expected, the number of interactions was dramatically increased in C8 and C9, ETA‐specific clusters, when compared with the common cluster of ETAs and ES‐aggregates at 48 h (C5) and 72–96 h (C2 and C10) (Figure 4F,G). We also identified the dominating interaction factors between the hTSCs and hESCs which were highly expressed in the ETA‐specific clusters, such as MK, PTN, FGF, ncWNT, WNT, and the 48 h specific factors DESMOSOME and GDF (Figure 4F,G).

To investigate the in vivo relevance, we compared all the ES‐derivates at 72–96 h with the scRNA‐seq data of a human gastrulating embryo at Carnegie state 7 (CS7).^31^ As the correlation analysis shows, the C10, a common cluster of 72–96 h ETAs and ES‐aggregates, was significantly similar to the primitive streak (PS) and the nascent mesoderm (NM) of in vivo embryo (Figure 4H). Consistently, the cells in C10 highly expressed the PS and the NM markers such as *TBXT*, *MIXL1*, and *EOMES* (Figure 4I). Moreover, we observed a large population of cells in C11 also expressed these PS‐ and NM‐specific markers (Figure 4I), however, the C11 cells did not exhibit a high correlation with PS, NM, or other PS cell derivates (Figure 4H). It is worth noting that the C11 was specific for ES‐aggregates and this type of cells with confusing fate was completely eliminated from the ES‐compartment of ETAs by co‐differentiation with hTSCs (Figure 4E). Collectively, we hypothesized a possible role for the extraembryonic trophectoderm in regulating the proper PS formation of the epiblast cells.

Next, we focused on the C8 which is a specific cluster for 72–96 h ETAs (Figure 4E). The cells in the C8 demonstrated a much stronger interaction between the ES‐compartment and the TS‐compartment (Figure 4G), which were negative for *TBXT*, and began to appear at 72 h and enriched at 96 h of ETAs, suggesting the C8 represents these undefined ETA‐specific flattened cells (Figure 3A) described above. To identify the C8, we compared C8 with the scRNA‐seq dataset of CS 8–11 monkey embryo.^32^ We found that C8 showed highest similarity to definitive endoderm (DE) and extra‐embryonic mesoderm cells (EXMCs) (Figure 4L). Considering the cells in C8 were derived from the pluripotent epiblast, and negative for the key endoderm markers *GATA6*, *SOX17*, and *AFP* (Figure 4I), thus we speculated the C8 cluster as EXMC‐like cells. To further characterize the C8, we compared the C8 with the scRNA‐seq data from naive ES and ES‐differentiated EXMCs. We found C8 was relatively similar to the early EXMCs, suggesting that hTSCs may facilitate the differentiation of epiblast into EXMCs (Figure 4K). GO analysis indicated that C8 had a higher expression of antiviral‐related terms compared with other ES‐derived cells in ETAs (Figure 4J). Taken together, these results suggested that trophectoderm regulated the EXMCs differentiation from the epiblast.

## 4 DISCUSSION AND CONCLUSION

In recent years, the 3D models generated using stem cells have greatly advanced our understanding of early mammalian embryo development. Several signals from extra‐embryonic tissues were found to be important for the remodelling of epiblast using the model constructed by mouse or human embryonic stem cells, such as the WNT, NODAL and BMP. In mouse, the embryo‐like structures constructed by assembling embryonic‐ and extra‐embryonic stem cells have provided the models for investigating the crosstalk between all three lineages (epiblast, primitive endoderm, and trophectoderm).

In this study, we introduced a 3D co‐culture system (ETAs) aggregating the hTSCs derived from human blastocysts, representing the development of trophectoderm, and the primed hESCs, representing the development of post‐implantation human epiblast. Compared with the ES‐aggregates cultured in the ETA system, both the ES‐aggregates and the ES‐compartments of ETAs undertook lumenogenesis and gave rise to T‐positive cells. Notably, the most of T‐positive cells in the ES‐compartments of ETAs were distributed at the distal region to the TS‐compartment, while, the T‐positive cells were randomly distributed in the whole epithelium of the ES‐aggregates, revealing an essential role of extraembryonic trophectoderm in asymmetric differentiation of human epiblast. Moreover, scRNA‐seq analysis indicated that the composition of T‐positive cells between ETAs and ES‐aggregates was largely different. The T‐positive cells in ETAs were more homogeneous and purely correlated with the in vivo PS and NM cells, the correct cell types they were supposed to differentiate into. In contrast, the T‐positive cells in ES‐aggregates showed high divergence and contained a large population of unidentified cells which not correlated with PS or any reported PS‐derivates, although they highly expressed the PS markers *TBXT*, *MIXL1*, *EOMES*, and so on. Our findings proposed a potential role of trophectoderm in regulating the proper PS formation of the human epiblast.

On the other hand, the origin of EXMCs in primates remains controversial and not well‐established. It was first thought that the EXMCs in primate embryos were derived from the extraembryonic endoderm, while some recent works have indicated that the epiblast, not the endoderm, is responsible for EXMCs generation in both cynomolgus monkey embryos^29^ and in vitro cultured hESCs,^33^ or the EXMCs in primates have hypoblast and epiblast dual‐origins.^32^ Here we observed a type of cell with a flattened morphology that lined aside the contact surface between the hESCs and hTSCs in 72–96 h ETAs. These cells were differentiated from the hESCs and transcriptionally similar to the monkey EXMCs^32^ and in vitro differentiated early human EXMCs.^33^ Our results provided new evidence for the epiblast‐origin of human EXMCs and raised a hypothesis that the EXMCs formation from human epiblast was induced by trophectoderm. However, further work is needed to validate the molecular regulatory mechanism of trophectoderm on EXMCs induction from the human epiblast.

In conclusion, the structure constructed in this study by aggregating hESCs and hTSCs could well simulate the asymmetric morphology and cell differentiation of human epiblast, and we provided a novel insight into the potential role of trophectoderm on the differentiation of epiblast to PS and EXMCs.

## AUTHOR CONTRIBUTIONS

Leqian Yu, Fan Guo, and Hongmei Wang initiated and supervised the study. Xulun Wu, Wentao Zhao, Xiaoyan Liang, and Kunyuan Yu performed the ETA‐related experiments, including immunostaining, imaging, and analyses. Yiming Wang and Xiaoyan Liang generated the hTSC line and performed the characterization. Qiancheng Zhang, Meijiao Wang, Hao Wu, Xulun Wu, and Wentao Zhao performed scRNA‐seq experiments and analysed data. Guang‐Hui Liu, Baoyang Hu, and Fan Mo provided hESCs and guided the culturing of hESCs. Shiwen Li and Xili Zhu guided the use of confocal laser‐scanning microscopes. Hongmei Wang, Jun Wu, Guang‐Hui Liu, Baoyang Hu, and Jinglei Zhai provided instructive suggestions and revised the manuscript. Leqian Yu, Xulun Wu, Hao Wu, Wentao Zhao, Qiancheng Zhang, Fan Guo, and Hongmei Wang analysed data and wrote the manuscript with inputs from other authors.

## FUNDING INFORMATION

We are grateful to Qi Zhou (CAS) for the instructive and helpful suggestions. This work was supported by the Strategic Priority Research Program of the Chinese Academy of Sciences (no. XDA16020700), the National Key Research and Development Program of China (nos. 2021YFA0805701 and 2022YFA1103101), the National Natural Science Foundation of China (no. 82192870), and by grants from CAS (no. ZDBS‐ZRKJZ‐TLC001).

## CONFLICT OF INTEREST STATEMENT

The authors declare no conflict of interest.

## Data Availability

The data that support the findings of this study are available from the corresponding author upon request.
