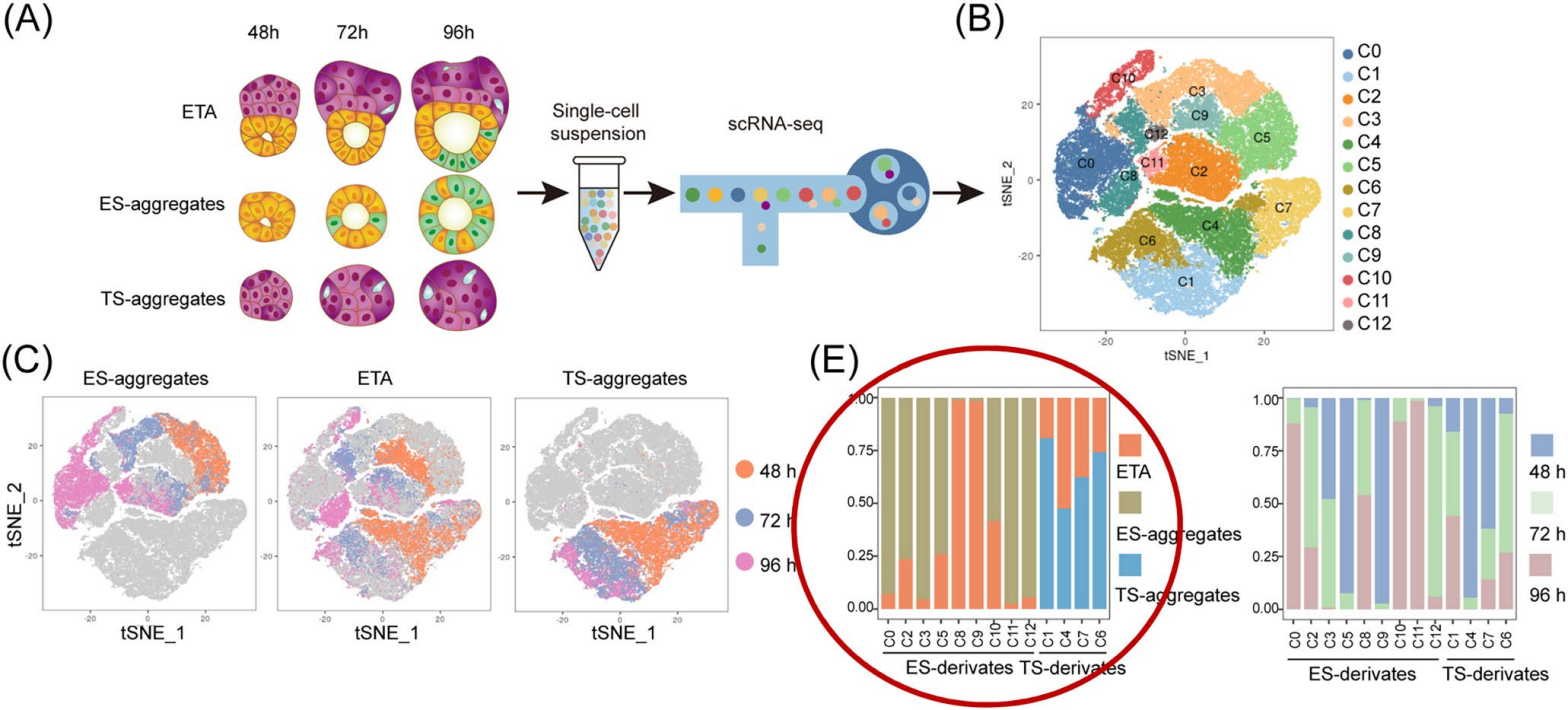# Correction to “An Aggregation of Human Embryonic and Trophoblast Stem Cells Reveals the Role of Trophectoderm on Epiblast Differentiation”

**DOI:** 10.1111/cpr.70115

**Published:** 2025-08-13

X. Wu, W. Zhao, H. Wu, Q. Zhang, Y. Wang, K. Yu, J. Zhai, F. Mo, M. Wang, S. Li, X. Zhu, X. Liang, B. Hu, G. H. Liu, J. Wu, H. Wang, F. Guo, and L. Yu, “An Aggregation of Human Embryonic and Trophoblast Stem Cells Reveals the Role of Trophectoderm on Epiblast Differentiation,” *Cell Proliferation* 56, no. 5 (2023 May): e13492, https://doi.org/10.1111/cpr.13492.

In the originally published version of this article, **Figure 4E** contained an unintended duplication error. Specifically, the left and right panels were mistakenly presented as identical due to a manual error during figure reformatting. This occurred while the figure was being adjusted for higher resolution.

We apologize for this error.

Originally published version of Figure 4E:

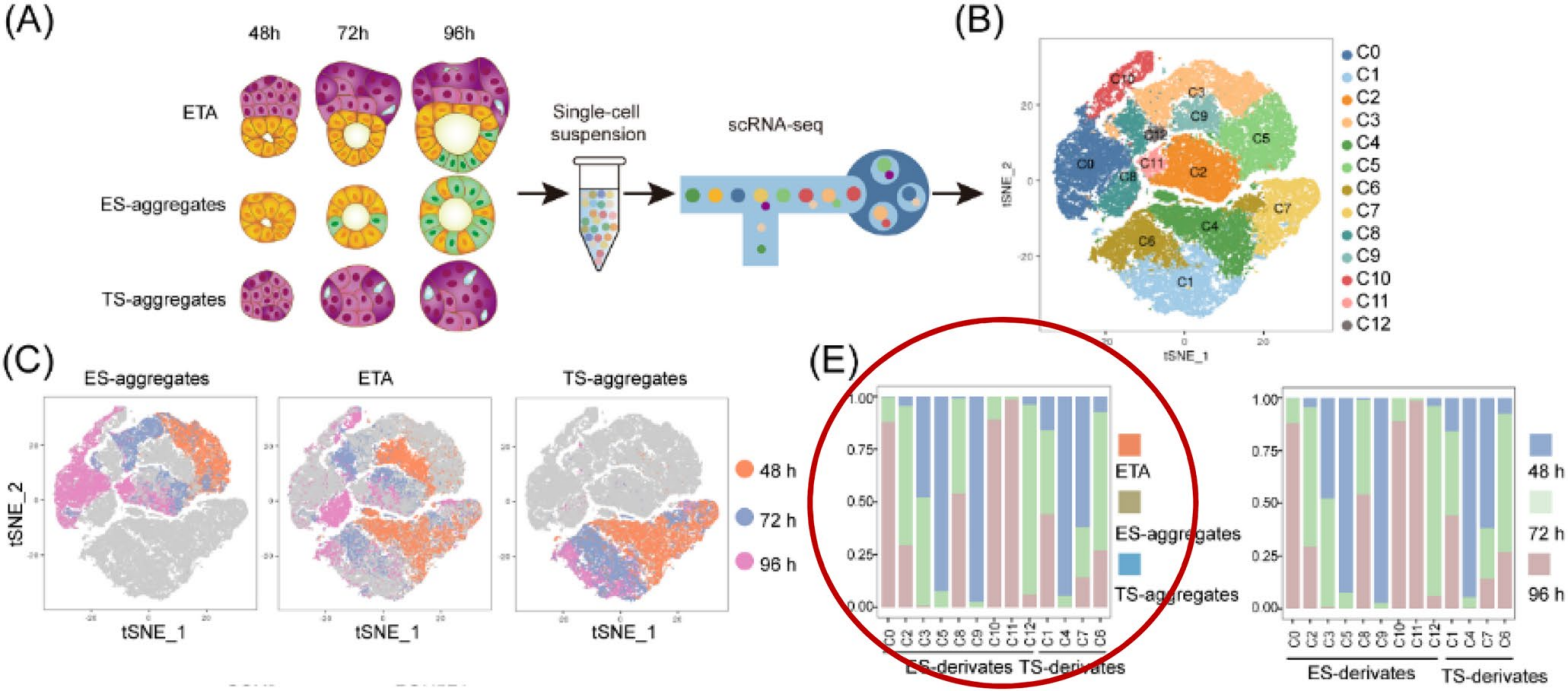

Corrected version of Figure 4E: